# Evaluation of Cellular Responses for the Diagnosis of Allergic Bronchopulmonary Mycosis: A Preliminary Study in Cystic Fibrosis Patients

**DOI:** 10.3389/fimmu.2019.03149

**Published:** 2020-02-07

**Authors:** Moïse Michel, Carine Gomez, Youssouf Sereme, Marion Gouitaa, Céline Chartier, Patricia Blanchard, Simon Pinchemel, Carole Cassagne, Stéphane Ranque, Jean-Louis Mège, Martine Reynaud-Gaubert, Joana Vitte

**Affiliations:** ^1^Aix-Marseille Univ, IRD, APHM, MEPHI, IHU Méditerranée Infection, MEPHI, Marseille, France; ^2^Medical Office, Marseille, France; ^3^Aix-Marseille Univ, APHM, Clinique des bronches allergies et sommeil, Marseille, France; ^4^APHM, IHU-Méditerranée Infection, UF Immunologie, Marseille, France; ^5^Aix-Marseille Univ, IRD, APHM, IHU Méditerranée Infection, VITROME, Marseille, France; ^6^Aix-Marseille Univ, APHM, Hôpital Nord, Service de pneumologie, Centre de Ressources et de Compétences en Mucoviscidose (CRCM) adulte, Marseille, France; ^7^AllergoBioNet Network, France

**Keywords:** basophil activation test, lymphocyte stimulation test, allergic mycosis, cellular tests, cystic fibrosis

## Abstract

**Background:** Allergic bronchopulmonary mycosis (ABPM) is an underestimated allergic disease due to fungi. Most reported cases are caused by *Aspergillus fumigatus* (Af) and are referred to as allergic bronchopulmonary aspergillosis (ABPA). The main risk factor of ABPA is a history of lung disease, such as cystic fibrosis, asthma, or chronic obstructive pulmonary disease. The main diagnostic criteria for ABPA rely on the evaluation of humoral IgE and IgG responses to Af extracts, although these cannot discriminate Af sensitization and ABPA. Moreover, fungi other than Af have been incriminated. Flow cytometric evaluation of functional responses of basophils and lymphocytes in the context of allergic diseases is gaining momentum.

**Objectives:** We hypothesized that the detection of functional responses through basophil and lymphocyte activation tests might be useful for ABPM diagnosis. We present here the results of a pilot study comparing the performance of these cellular assays vs. usual diagnostic criteria in a cystic fibrosis (CF) cohort.

**Methods:** *Ex vivo* basophil activation test (BAT) is a diagnostic tool highlighting an immediate hypersensitivity mechanism against an allergen, e.g., through CD63 upregulation as an indirect measure of degranulation. Lymphocyte stimulation test (LST) relies on the upregulation of activation markers, such as CD69, after incubation with allergen(s), to explain delayed hypersensitivity. These assays were performed with Af, *Penicillium*, and *Alternaria* extracts in 29 adult CF patients.

**Results:** BAT responses of ABPA patients were higher than those of sensitized or control CF patients. The highest LST result was for a woman who developed ABPA 3 months after the tests, despite the absence of specific IgG and IgE to Af at the time of the initial investigation.

**Conclusion:** We conclude that basophil and lymphocyte activation tests could enhance the diagnosis of allergic mycosis, compared to usual humoral markers. Further studies with larger cohorts and addressing both mold extracts and mold relevant molecules are needed in order to confirm and extend the application of this personalized medicine approach.

## Introduction

Molds are microscopic fungi ubiquitous in the environment. In immunosuppressed patients, they cause localized or systemic infections. What is less well-known outside allergy clinics is that molds are frequent airborne sensitizers involved in allergic diseases, the most frequent and life-threatening being allergic bronchopulmonary mycosis (ABPM). Most reported cases are attributed to *Aspergillus fumigatus* (Af), which are referred to as allergic bronchopulmonary aspergillosis (ABPA). ABPA occurs in patients with a history of chronic lung disease, such as cystic fibrosis (CF), asthma, or chronic obstructive pulmonary disease (1). The current hypothesis is that chronic inflammatory bronchial diseases alter the immune responses by triggering a Th2 immune response instead of an efficient immune clearance following contact with molds (2, 3). Despite several diagnostic criteria updates, the main criterion still relies on the evaluation of humoral IgE and IgG responses to Af extracts, with the shortcoming that these cannot discriminate Af sensitization from ABPA (4). The determination of IgE responses to Af individual proteins with proven allergenicity, commonly referred to as “molecular allergens,” improves ABPA diagnostic accuracy (5, 6). Yet, although ABPM was firstly described and most frequently associated with Af, other molds have been documented to trigger allergic pulmonary disease. Their diagnostic criteria are poorly defined and they are infrequently reported in the literature.

The evaluation of the functional cellular responses against allergens is a diagnostic criterion that is currently used in international guidelines (7, 8). *Ex vivo* basophil activation test (BAT) investigates immediate hypersensitivity events whereas lymphocyte stimulation test (LST) explores delayed hypersensitivity. In both tests, whole blood is incubated with serial concentrations of a suspected allergen, followed by flow cytometric quantification of upregulated activation markers (9, 10). Data are scarce for mold-related allergic diseases (11–13). This study aimed to assess the relevance of functional cellular assays for ABPM diagnosis and review *ex vivo* basophil and lymphocyte functional cellular tests in ABPM diagnosis.

## Patients and Methods

### Patients

Adult patients (*n* = 29) followed in our French cystic fibrosis care center and the lung diseases department (Assistance Publique—Hôpitaux de Marseille) were routinely assessed for ABPM diagnosis between April 2017 and January 2018. Patients were categorized in accordance with the final diagnosis as ABPA, Af-sensitized (AF-S), fungal colonization, or control CF patients. These categories were defined as follows: ABPA met all the ISHAM criteria (4); AF-S displayed sIgE to Af (0.1 kUA/L or greater) without fulfilling the ISHAM criteria for ABPA; fungal colonization was defined as at least one filamentous fungus cultured from a bronchial sample during the previous 6 months, without fulfilling the ISHAM criteria, while patients who were categorized in none of the previous categories were considered as control CF patients. Demographic and laboratory data for the study cohort are detailed in Table 1.

**Table 1:** Demographic and laboratory findings of the study cohort.

	**Control**	**Af-sensitized**	**ABPA**	**Fungal** **colonization**	**Total**
*n*	16	3	3	7	29
Age (years) (median ± 5–95 percentile)	43 (26-61)	35 (27-44)	21 (17-24)	42 (22-62)	38 (18-62)
Male/female	7/9	1/2	0/3	5/2	13/16
Lung transplantation	12/16	3/3	0/3	4/7	19/29
Time since transplantation (median ± 5–95 percentile)	6 (1-19)	7 (4-14)	/	2 (1-10)	6 (1-17)
Bacterial colonization	9/16	1/3	3/3	6/7	19/29
Fungal colonization	7/16	2/3	3/3	6/7	18/29
Chest HRCT abnormality(ies)	14/16	2/3	3/3	7/7	26/29
Total IgE (kIU/L) (median ± 5–95 percentile)	73 (4–333)	72 (41–100)	414 (74–854)	12 (2–26)	94 (2–469)
IgE Af (kUA/L) (median ± 5–95 percentile)	<0.1	1 (0.1–2)	10 (3–19)	<0.1	1 (0–6)
Eosinophils (mm^3^) (median ± 5–95 percentile)	321 (107–623)	327 (161–602)	370 (135–775)	319 (149–565)	326 (128–692)

*ABPA, Allergic bronchopulmonary aspergillosis; Af, Aspergillus fumigatus; HRCT, High-resolution computed tomography*.

### Functional Cytometric Tests

All the assays were done with Af, *Penicillium notatum* (Pen), and *Alternaria alternata* (Alt) extracts (Bühlmann Laboratories®, Schönenbuch, Switzerland). BAT was performed with the Flow2CAST method (Bühlmann Laboratories®), using CCR3 (CD193) and CD63 as basophil identification and activation markers, following the manufacturer's instructions. Positive controls were anti-RFcεI and the bacterial peptide fMLP. For LST, whole blood was incubated in a 96-well plate with RPMI 1640 medium (Thermo Fisher Scientific, Waltham, MA) and sequential allergen dilution for 24 h under 5% CO_2_. Phytohemagglutinin (PHA, Thermo Fisher Scientific), 10 μg/L, was used as a positive control. Each well was harvested and stained with a mix of the following antibodies: PerCP-anti-CD45 (clone 2D1), FITC-anti-CD3 (clone SK7), APC-anti-CD4 (clone SK3), PE-anti-CD8 (clone SK1), and PeCy7-anti-CD69 (clone L78) (BD Biosciences®, San Diego, California). Flow cytometry was performed on a FACS Canto II (Becton Dickinson, Le Pont de Claix, France) and at least 200 basophils per sample were analyzed for BAT and 10,000 lymphocytes for LST.

### Data Expression

Data were analyzed using FACS Diva software (TreeStar, Ashland, OR). Specific IgE (sIgE) to Af extract levels were measured with the Thermo Fisher ImmunoCAP platform (Phadia, Thermo Fisher Scientific, Uppsala, Sweden). All the results were expressed as the basophil or lymphocyte stimulation index, which is the ratio between level of activation with the allergen and level of activation with reaction buffer, with a threshold of 2. Statistical analysis was performed with the R statistical software (14). A correlation matrix was calculated using Pearson's correlation. Mean responses of each group were compared via the Student or Kruskal–Wallis test, a two-sided *p* < 0.05 was statistically significant.

### Ethics Statement

The study was based on a retrospective review of medical charts and laboratory results. Under the French law, ethics committee approval and patient consent were not required for this type of non-interventional study, provided the patients had received information and retained the right to oppose the use of anonymized medical data (15, 16).

## Results

The results of two patients were excluded from the analysis: one patient with an absolute basopenia (excluded from BAT analysis) and one patient with a deep lymphopenia (<300/mm^3^, excluded from LST analysis). Negative and positive controls were acceptable for BAT (median 5.19%, range 4.16–5.50, and 85.92%, range 44.99–97.85, respectively) and LST [median 1.52% (1.10–1.97) and 65.39% (31.81–86.89) for CD8 LST, mean 1.29% (1.00–1.80) and 68.32% (45.89–85.73) for CD4 LST]. Status of lung transplantation did not influence the level of basophil activation in anti-RFcεI positive control (median 18.64, range 6.48–21.23 and median 18.54, range 11.96–21.90, *p* = 0.53, for transplanted and non-transplanted patients, respectively) and with all the mold extracts (median 1.68, range 0.69–4.28 and median 1.35, range 0.62–2.11, *p* = 0.31 for Pen; 1.35, range 0.66–6.71 and 1.38, range 0.77–20.37, *p* = 0.07 for Af; 1.29, range 0.60–4.48 and 1.27, range 0.72–7.77, *p* = 0.44 for Alt).

**Figure 1:**
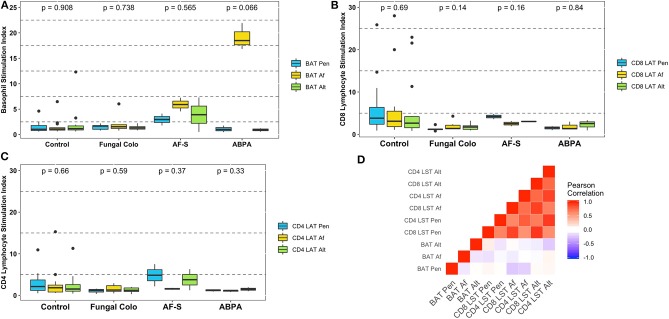
Results of the functional cytometric tests with mold extracts expressed as the stimulation index: BAT results **(A)**, T CD8 LST results **(B)**, T CD4 LST results **(C)**, and correlation matrix of functional cytometric tests with mold extracts **(D)**. *p-*value of the Kruskal–Wallis test, which compares results from Pen, Af, and Alt extracts, is written above each group of patients. ABPA, Allergic bronchopulmonary aspergillosis; Af, *Aspergillus fumigatus* extract; AF-S, Af-sensitized patients; Alt, Alternaria extract; BAT, Basophil activation test; Control, Control patients (without any *Aspergillus*-related disease); Fungal Colo, Fungal colonized-patients; LST, Lymphocyte stimulation test; Pen: *Penicillium* extract.

Figure 1A shows that 15 patients (56%) had a positive BAT with at least one mold extract: 10 BAT Af, 6 BAT Pen, and 4 BAT Alt positive. Three patients and one patient had a positive BAT with two or all the three extracts, respectively. BAT showed low levels of activation in controls, in Af-sensitized patients, and in mold-colonized patients. The ABPA group, in which no patient was not lung transplanted, responded with higher basophil activation to Af as compared to other groups, but statistical significance was not reached due to the small sample size (three patients, *p* = 0.066). Af-induced basophil activation in these patients was higher than responses to Pen and Alt (mean 18.48, range 17.00–21.58 for Af vs. 1.02, range 0.54–1.69 for Pen and 0.97, range 0.60–1.21 for Alt), although the significance level was not reached either (*p* = 0.066). These patients had no positive responses with Pen and Alt extracts. When specific IgE to Af was positive (>0.10 kUA/L), BAT Af was significantly higher than BAT Pen and Alt (*p* = 0.008).

Figures 1B,C describes results of lymphocyte activation, which was an infrequent finding with any mold extract and in all patient groups: mean for CD8 LST 3.40 (0.99–7.31), 3.26 (2.84–3.67), 1.87 (1.12–2.67), and 1.82 (1.04–3.38); mean for CD4 LST 1.71 (0.57–3.17), 3.39 (1.76–5.02), 1.23 (0.84–1.49), and 1.50 (0.50–2.69) in control, AF-S, ABPA, and fungal colonization groups, respectively.

Levels of CD4 and CD8 LST did not differ as a function of lung transplantation status or with the level of specific IgE to Af (data not shown). However, the only patient who displayed a strongly positive CD8 and CD4 LST with all the extracts is worth of notice. This 29-year-old woman, lung transplanted with no detectable sIgE to Af presented during the initial investigation with pulmonary micronodules, mucus plugging, and ground glass appearance (thickening and impaction of bronchioles) as evidenced by high-resolution computed tomography chest. Despite this evocative presentation, ABPA was not diagnosed because of the absence of humoral IgE and IgG responses to Af. However, she developed an ABPA 3 months after.

### Correlation Analysis

The correlation matrix at Figure 1D showed a strong positive correlation of both CD4 and CD8 responses to each mold extract (correlation coefficient *r*-values ranging from 0.56 to 0.92, *p* < 0.001). In contrast, neither basophil responses to distinct mold extracts nor basophil and lymphocyte responses to a given mold extract were statistically significantly correlated (*r*-values ranging from −0.24 to 0.09).

## Discussion and Review

Fungal species demonstrated as triggers of allergic pulmonary diseases have been reviewed previously (17, 18), while cellular assays for ABPM diagnosis were reported as early as 1977, as described in Table 2 (19–22, 25). BAT can demonstrate an immediate-hypersensitivity mechanism (8, 9). Only three studies have evaluated the utility of BAT for ABPA diagnosis so far. All focused on CF patients, a background associated with the highest incidence of ABPA (26). Mirković et al. showed that BAT with Af extract identified sensitized patients and suggested that a combination of BAT and routine workup could detect ABPA. Moreover, the level of basophil activation was correlated with decreased lung function tests, suggesting that BAT could be used not only as a diagnostic assay, but also as a prognostic marker. Gernez et al. confirmed BAT as an effective and robust diagnostic assay for ABPA, although in their hands, in a contradictory manner, it could not discriminate between Af-sensitized and ABPA CF patients. Katelari et al. proposed the BAT cutoffs of 60.30 and 76.86% of CD63 and CD203c basophil activation for ABPA diagnosis with good sensitivity and specificity. In our patients, BAT with Af extract discriminated Af sensitized from ABPA patients by using a higher threshold. Thus, BAT with fungal antigens is a promising diagnostic tool for ABPM, but further studies are needed to prove the suitability in non-CF patients and by using other fungal antigens.

**Table 2:** Overview of reports on cellular assays as a diagnostic tool for allergic mycosis.

**References**	**Population**	**Allergen(s)**	**Markers**	**Main conclusions**
Hollmann et al. (19)	79 *C. albicans*-colonized patients 30 healthy controls	*C. albicans*	LST with ^3^H-thymidine	57% of *C. albicans*-colonized patients show lymphocyte response
Brunet et al. (20)	60 *C. albicans*-delayed HS patients	*C. albicans*	LST with CD25 and CD69	All the patients have CD25 T cells Only patients with syndromic reactions have CD69 T cells
Tsushima et al. (21)	7 patients	*L. aggregatum*	LST with ^3^H-thymidine	LST is positive in peripheral blood and BAL for all patients Diagnosis of HS pneumonitis induced by *L. aggregatum*
Yoshikawa et al. (22)	One case report	*P. citrinum*	LST with ^3^H-thymidine	LST is positive in peripheral blood and BAL Diagnosis of HS pneumonitis induced by *P. citrinum*
Matsuno et al. (23)	One case report	*A. alternata* *A. fumigatus* *C. albicans*	LST with ^3^H-thymidine Evaluation of IL-5 production	LST are positive with *A. fumigatus* and *C. albicans* Only *C. albicans* culture produce IL-5 *in vitro* Diagnosis of acute eosinophilic pneumonia caused by *C. albicans*
Luong et al. (24)	10 allergic fungal rhinosinusitis 11 healthy controls	*A. alternata* *A. fumigatus* *C. herbarum* *P. notatum*	LST with evaluation of cytokine production after 72 h of incubation	Fungal antigens stimulate T-cell activation, with a Th2 immune response (IL-4 and IL-5 production)
Ogawa et al. (25)	Two case reports	*S. commune*	LST with ^3^H-thymidine	LST is positive in peripheral blood Diagnosis between *Schizophyllum*-asthma and ABPM
Mirkovic et al. (12)	48 CF patients 11 healthy controls	*A. fumigatus*	BAT with CD203c	BAT discriminates non-sensitized and Af-sensitized patients BAT is inversely correlated with FEV1 Antifungal therapy does not altered BAT results
Gernez et al. (11)	74 CF patients 2 asthmatic patients	*A. fumigatus* Asp f 1	BAT with CD203c and CD63	CD203c shows better discriminated performance than CD63 BAT with Af discriminates ABPA and no-ABPA CF patients BAT with Af does not discriminate non-sensitized and Af-sensitized patients BAT with Asp f 1 discriminates ABPA and no-ABPA CF patients
Katelari et al. (13)	56 CF patients	*A. alternata* *A. fumigatus*	BAT with CD203c and CD63	BAT with Af discriminates ABPA and no-ABPA CF patients with a high threshold BAT with Af discriminates Af-sensitized patients who run a higher risk of ABPA No correlation between BAT with Af and BAT with A. alternata

*A. alternata, Alternaria alternata; ABPM, Allergic bronchopulmonary mycosis; B. adusta, Bjerkandera adusta; BAL, Bronchoalveolar lavage; C. albicans, Candida albicans; C. herbarium, Cladosporium herbarum; CF, Cystic fibrosis; FEV1, Forced expiratory volume in 1 s; HS, Hypersensitivity; L. aggregatum, Lyophyllum aggregatum; P. citrinum, Penicillium citrinum; P. notatum, Penicillium notatum; S. commune, Schizophyllum commune*.

Evaluation of the lymphocyte responses to allergen is a useful delayed drug hypersensitivity diagnostic tool (10, 27–29). LST has long been used as a diagnostic tool for delayed hypersensitivity against several fungi (Table 2). The incorporation of tritiated thymidine was the gold standard for the detection of fungal-specific T cells. Owing to the constraints inherent to radioactive methods, new markers were developed, with CD69 and CD25 upregulation being the most popular (30–32). Currently, an alternative is the lymphocyte proliferation test with 5,6-carboxylfluorescein diacetate succinimidyl ester (CFSE) (33). Few studies have evaluated T helper (Th) 2 cytokine production after fungal stimulation, notably IL-5, for ABPM diagnosis (23, 24). Stimulation with Af extract and recombinant proteins in ABPA patients has shown an unusual Th2 immune response (34). Patients with invasive aspergillosis displayed a Th1 phenotype (35) and a Th17 phenotype (36), corresponding to a relevant anti-infectious immune activation. Taken together, these studies show that the immune skew present in a given patient's responses to fungal antigens can be evidenced through functional tests. However, the specificity of Th2 activation induced by fungal antigens as a diagnostic marker for ABPM has not been established, restricting the relevance of this method for ABPM diagnosis. Recently, a new method was developed, based on magnetic antigen-reactive T cell enrichment (ARTE) of CD154^+^ (CD40L^+^) T cells, which allowed the identification of rare populations of antigen-specific T cells. Briefly, after PBMC and antigen coculture, antigen-specific cells were sorted via magnetic beads, separating CD154+ conventional T (Tcon) cells from CD137^+^ CD154^−^ regulatory T cells. Magnetic enrichment allowed an easy and detailed flow cytometry analysis of subpopulations (37). Tcon cells from lung immunocompromised patients showed a strong Th2 activation after fungal antigen stimulation (38). In our study, LST was not performant for ABPM diagnosis. Yet, our results suggest that LST might predict the development of ABPM. Indeed, the patient with the highest results in our cohort, presenting with a strong CD4 and CD8 T cell activation to fungal extracts, developed an ABPA 3 months after the abnormal LST assay. Detection of peripheral fungal-specific T cell, which can activate downstream humoral responses, may highlight a pathological process underlying subclinical ABPA with the potential of development of full-blown ABPA. A larger study with an extended follow-up will be essential to confirm this hypothesis.

*Ex vivo* cellular activation against several fungi also suggests that ABPM is the result of molecular epitope spreading due to similar T cell activation against three distinct fungi. Watai et al. showed that *de novo* sensitization to fungal antigens is constant during life, contrary to most inhalant allergens (39). Our data showed a major correlation in LST results with all the three allergens, thus an LST cross-reactivity due to a permanent sensitization addition on the T cell repertoire in CF patients.

## Conclusion

Functional cellular assays are emerging biomarkers for the diagnosis of allergic mycoses. Whereas, IgE and IgG sensitization, which may result from a normal contact with airborne environmental molds, are indirect biomarkers with insufficient specificity, the *ex vivo* functional cellular activity is a direct marker of *in vivo* mechanisms. Because basophils are involved in lung tissue damage, a strong *ex vivo* basophil activation might not only be an indirect marker of an IgE-linked immune reaction, but also a direct marker of lung disease. Conversely, because T lymphocytes are involved in the initiation, development, and maintenance of the lung Th2-immune response, a major *ex vivo* T cell activation might occur in patients with sub-clinical disease that possibly would progress to overt ABPM. The major weakness of our study is the low number of ABPA cases, due to the prospective design. The major strength is the investigation by means of innovative diagnostic tools.

## Data Availability Statement

All datasets generated for this study are included in the article/supplementary material.

## Ethics Statement

The study was based on a retrospective review of medical charts and laboratory results. Under the French law, ethics committee approval and patient consent were not required for this type of non-interventional study, provided the patients had received information and retained the right to oppose the use of anonymized medical data (15, 16).

## Author Contributions

MM, CG, MR-G, and JV contributed conception and design of the study. CG performed the experiments. YS and CCh organized the database. MG, PB, and SP performed the statistical analysis. MM wrote the first draft of the manuscript. CCa, SR, and J-LM wrote sections of the manuscript. All authors contributed to manuscript revision, read and approved the submitted version.

### Conflict of Interest

The authors declare that the research was conducted in the absence of any commercial or financial relationships that could be construed as a potential conflict of interest.
